# Supplementary material for: LRP1-Mediated AggLDL Endocytosis Promotes Cholesteryl Ester Accumulation and Impairs Insulin Response in HL-1 Cells
Source: Cells. 2020 Jan 10;9(1):182. doi: 10.3390/cells9010182 (PMC7016900; doi:10.3390/cells9010182)
Supplement: Supplementary file 1 [file cells-09-00182-s001.pdf]

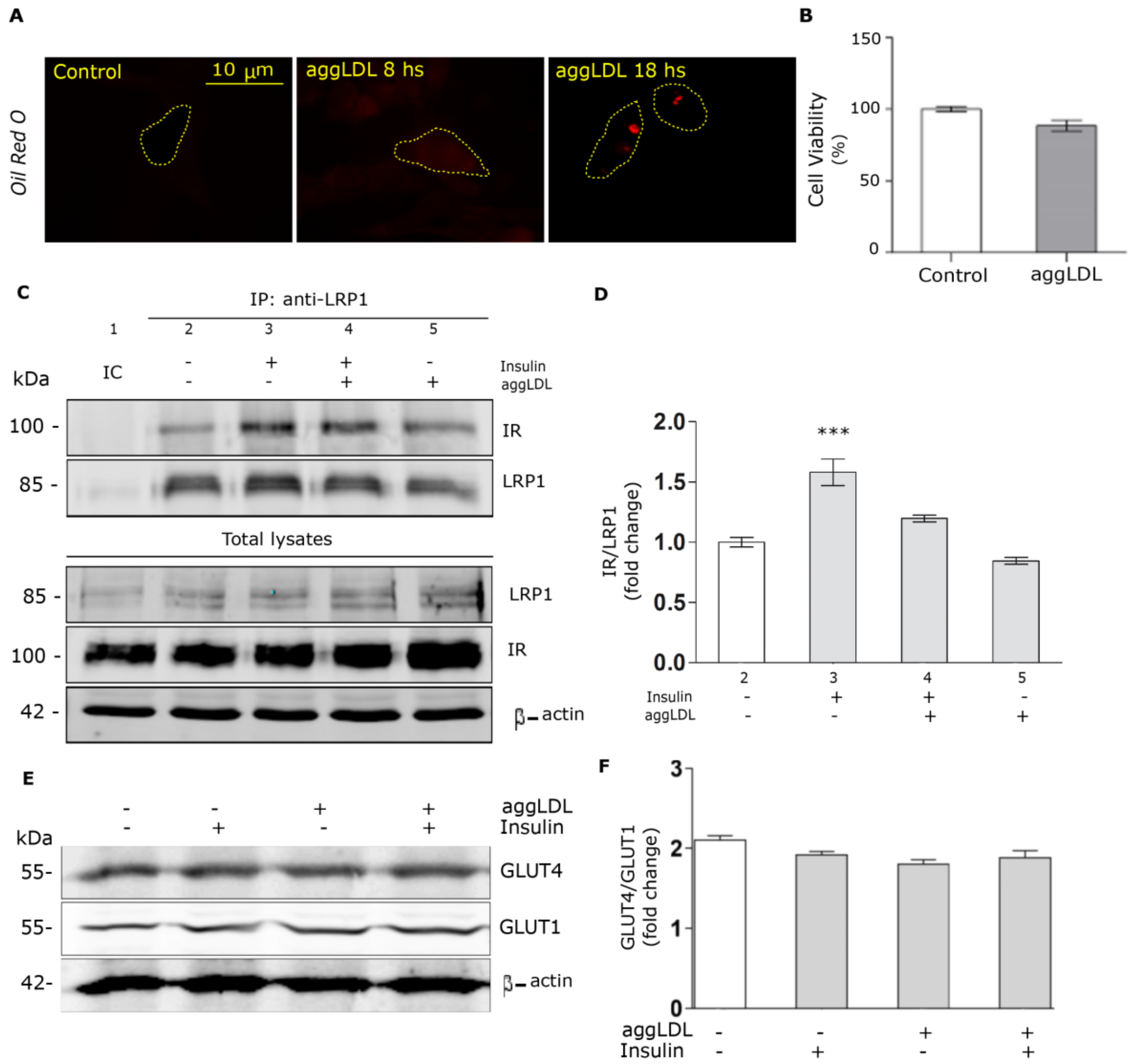

Suppl. Fig. S1

Figure S1. Additional experiments and data. A) Optical microscopy assay for detection of lipid droplets (LD) in HL-1 cells treated or not with 100 µg/ml aggLDL for 8 and 18 h, subsequently stained with *Oil Red O*. Then, they were thoroughly washed with 1X PBS and fixed with 4% paraformaldehyde for 15 min. Subsequently, three washes were carried out with 65% isopropanol and then incubated with a solution of *Oil Red O* (0.7 g in 200 ml of isopropanol) for 30 min. The dye solution was removed and washed immediately with 65% isopropanol and then with distilled water. Fluorescence images were obtained at 562 nm using the Leica DMI8 microscope. The yellow dotted line frames show the cell shape. The scale bar corresponds to 10 µm. Three independent experiments were performed by duplicate. **B)** Cellular viability assay in HL-1 cells incubated with 100 µg/ml aggLDL for 8 h. Cell viability was evaluated using the metabolic dye 3-(4,5-dimethlthiazol-2-yl)-2,5-diphenyl-tetrazolium bromide (MTT). Briefly, MTT solution (5 mg/ml) was added to the culture medium (1:10) and incubated for 2.5 h at 37 °C. After the incubation, the medium was removed and the precipitated dye was dissolved in 100 µl of DMSO. Absorbance was read at 540 nm, and the results were expressed as percentage cell viability relative to control condition. Three independent experiments were performed by triplicate. **C)** Immunoprecipitation assay for LRP1. Cell lysate was immunoprecipitated with an anti-LRP1 antibody and then subjected to SDS-PAGE and followed by Western blot assays for IR and total LRP1. Cells were incubated or not with 100 µg/ml aggLDL for 8 h and then stimulated or not with 100 nM insulin for 30 min. Lane 1, immunoglobulin isotype control. The detection of LRP1, IR and β-actin in total cell lysates is shown in the lower panel of the Western blot. **D)** Bar graph represents the densitometric quantification of the bands obtained in the Western blot. Values were

expressed as Mean ± SEM of the relative intensity of the detection of IR with respect to LRP1, as fold change, with respect to the control (white bar). (***) ($p < 0.0001$) indicates significant differences respect to control (white bar). Three independent experiments were performed by duplicate. **E)** Western blot assay of GLUT4 and GLUT1 in HL-1 cells treated with aggLDL as above and stimulated or not with 100 nM insulin for 30 min. The immunodetection of β-actin was used as a protein loading control. **F)** Bar graphs represent the densitometric quantification of the bands obtained in the Western blot results. Respective values were expressed as Mean ± SEM of the relative intensity of GLUT4/GLUT1 represented as fold change against the controls (white bar). Three independent experiments were performed by duplicate.
